# Severe recurrent *Streptobacillus moniliformis* endocarditis in a pregnant woman, and review of the literature

**DOI:** 10.1186/s13756-020-00789-4

**Published:** 2020-07-29

**Authors:** Kathryn R. Crofton, Jiqing Ye, Emil P. Lesho

**Affiliations:** 1 University of Rochester School of Medicine and Dentistry, Rochester, NY USA; 2 Department of Pathology, Rochester Regional Health, Rochester, NY USA; 3 Department of Infectious Diseases, Rochester Regional Health, 1425 Portland Ave, Rochester, NY 14621 USA

**Keywords:** Pregnancy, Pregnant, Rat bite fever, Endocarditis, *Streptobacillus moniliformis*

## Abstract

**Background:**

Rat bite fever is a systemic febrile illness caused by infection with the Gram-negative bacillus *Streptobacillus moniliformis* following a bite, scratch, or contact with excrement. Only 26 cases of native valve endocarditis have been reported to date. We could find no other reports of severe *Streptobacillus* endocarditis requiring valve replacement in a young, pregnant patient.

**Case presentation:**

A pregnant patient sought care for right leg pain, fevers, left upper quadrant pain, generalized weakness, fatigue, and inability to bear weight on her right leg. She had a syncopal episode 9 months earlier, resulting in a mandibular fracture and internal fixation hardware. Her pregnancy was complicated by hyperemesis and weight loss. Her pets included a rescued wild bird, a cat, and four rats. Her parents rescued stray cats, and she recalled multiple cat bites and scratches since childhood. She denied injection drug use. Ultrasound indicated a right popliteal artery thrombus. Transesophageal echocardiogram revealed a 2 cm × 0.7 cm vegetation. Angiography demonstrated multiple splenic infarcts and bilateral renal infarcts. She underwent mitral valve repair. The mitral valve Gram stain demonstrated 2+ Gram-negative rods, rare Gram-positive rods, and moderate white blood cells. *Propionibacterium* spp. was isolated from the mitral valve tissue on Columbia agar incubated anaerobically. Anaerobic and aerobic cultures of the valve tissue on all other broths and agars remained negative at 14 days. Hematoxylin and eosin stains showed a fibro-inflammatory vegetation. Aggregates of rod-shaped bacteria were identified on Warthin Starry/Steiner stain. *Bartonella* titers were positive for *B. henselae* IgG 1:256, IgM < 1:20. Brown-Hopps Gram stain, AFB, and GMS stains for bacterial and fungal microorganisms were negative. Broad range bacterial PCR and sequencing of a segment of 16 s rRNA gene of the valve tissue matched to *Streptobacillus* sp. (genus level) and most closely related to *Streptobacillus moniliformis*.

**Conclusion:**

This case demonstrates diagnostic and therapeutic challenges associated with a relatively uncommon cause of endocarditis. The diagnosis of rat bite fever was delayed due to symptoms of a concomitant pregnancy. Other confounders included possible alternative sources or co-infections with another zoonosis from multiple pets, and an odontogenic source due to presence of exposed jaw hardware.

## Background

Rat bite fever typically begins with a bite or other exposure, followed by abrupt onset of systemic illness, including intermittent relapsing fever, arthritis, and rash 3 days to 3 weeks later. A maculopapular, petechial, or purpuric rash develops in approximately 75% of those affected in the first symptomatic week [1, 2]. Over half of those affected develop migratory polyarthralgias, with involvement of both large and small joints of the extremities [1]. If untreated, the mortality rate approaches 10% [2]. Endocarditis has been described as a complication, but only 26 cases of native valve endocarditis have been reported to date [3]. We found no reports involving pregnant patients.

We sought to alert clinicians to the challenges and potential pitfalls in the diagnosis and management of recurrent *Streptobacillus* endocarditis in a pregnant patient. This case is noteworthy because it demonstrates diagnostic and therapeutic challenges associated with a relatively uncommon cause of endocarditis. For example, the typical symptoms of rat bite fever were masked by the symptoms of a concomitant pregnancy. Other diagnostic confounders included possible alternative sources or co-infections with another zoonosis from multiple exposures, and an odontogenic source due to presence of exposed jaw hardware. It is novel because we could find no other reports of severe *Streptobacillus* endocarditis requiring valve replacement in a young, pregnant patient.

## Case presentation

A previously healthy 24 year-old female, who was 13 weeks pregnant, sought care for 2 weeks of severe right leg pain and fevers (39.4 degrees C recorded at home), which progressed to bilateral calf and left upper quadrant pain, generalized weakness, fatigue, and inability to bear weight on her right leg. Her past medical history was notable for a syncopal episode 9 months earlier, resulting in a mandibular fracture and internal fixation hardware. Her pregnancy was complicated by hyperemesis and weight loss. Her pets included a rescued wild bird, a cat, and four rats. Her parents rescued stray cats, and she recalled multiple cat bites and scratches since childhood. She also allowed her rats to nibble on her fingers, most recently several weeks prior to admission. The patient also complained of exposed mandibular hardware. She denied injection drug use but used marijuana for nausea. She reported a history of rash when taking penicillin.

She was pale and had a temperature of 38.4 C, with diminished pedal and tibial pulses on the right. She had a 2/6 high pitched blowing holosystolic murmur, radiating to the axilla. She also had left upper quadrant tenderness and a palpable spleen. There were no rashes or lymphadenopathy. A metal plate was visible below the lower right teeth, with generally good dentition. The right calf was tender to palpation. A healing bite wound on her index finger was clean, without swelling or tenderness. Laboratory evaluation revealed iron deficiency anemia, normal renal function, normal hepatic enzymes, and a normal leukocyte count and differential.

An ultrasound indicated a right popliteal artery thrombus. Transesophageal echocardiogram revealed a 2 cm × 0.7 cm vegetation on the atrial side of the posterior mitral valve leaflet. Angiography demonstrated multiple splenic infarcts and bilateral renal infarcts.

**Fig. 1:**
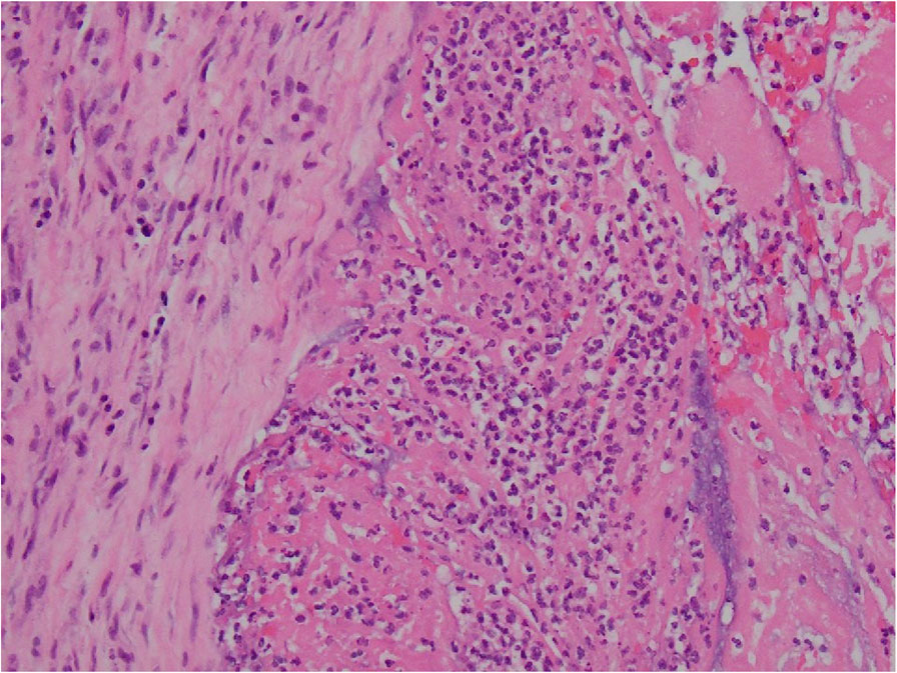
Hematoxylin and eosin stain of mitral valve tissue at 20x magnification showing fibroinflammatory vegetation

**Fig. 2:**
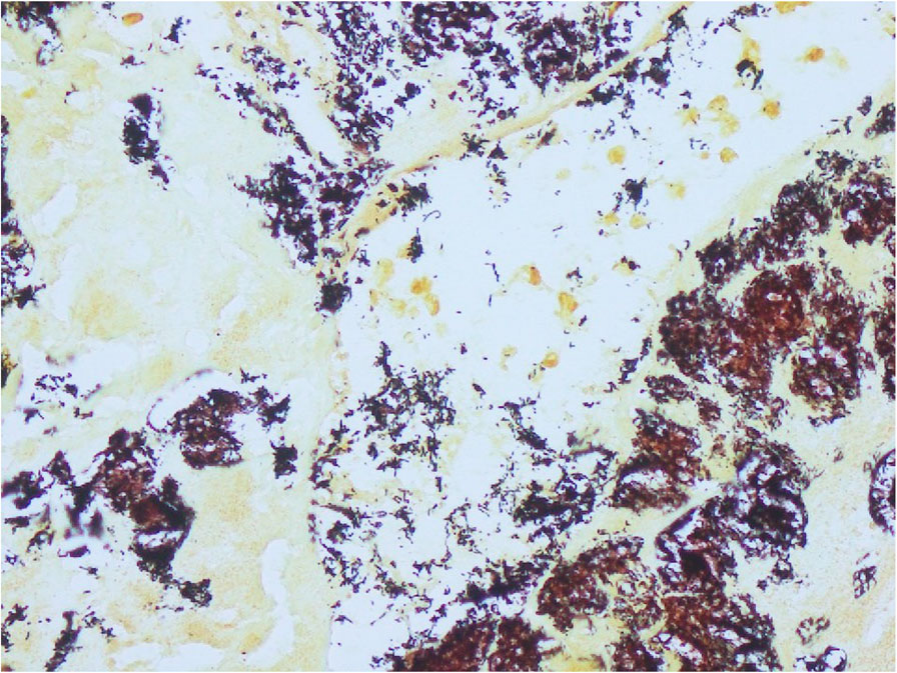
Warthin Starry stain showing aggregates of rod-shaped bacteria in the vegetation

Right popliteal thrombectomy was performed. Blood and popliteal thrombus cultures remained negative. The patient was treated empirically for *Streptobacillus moniliformis* given the history of rat bites and Streptococcus viridans/HACEK given history of oral surgery with exposed metal plates. The initial antibiotic regimen was ceftriaxone 2 g q12h and vancomycin 1.5 g q12h. Blood cultures and the popliteal thrombus did not initially grow any organisms, so clindamycin was added for empiric anaerobic coverage, as well as for *Streptobacillus moniliformis* (given the history of rat bites from her pet rats) and *Streptococcus viridans*/HACEK (given her history of oral surgery and exposed intra-oral hardware). On hospital day 4, she underwent mitral valve repair using a 27 mm band. The mitral valve Gram stain demonstrated 2+ Gram-negative rods, rare Gram-positive rods, and moderate white blood cells. *Propionibacterium spp.* was isolated from the mitral valve tissue on Columbia agar incubated anaerobically. Anaerobic and aerobic cultures of the valve tissue on all other broths and agars, including Chocolate, charcoal yeast extract, and serum-supplemented, remained negative at 14 days. Hematoxylin and eosin stains showed a fibroinflammatory vegetation (Fig. 1) and aggregates of rod-shaped bacteria were identified on Warthin Starry/Steiner stain, (Fig. 2). *Bartonella* titers were strongly positive for *B. Henselae* IgG 1:256, IgM < 1:20. Brown-Hopps Gram stain, AFB, and GMS stains for bacterial and fungal microorganisms were negative. PCR and 16 s rRNA sequencing of MV tissue identified *Streptobacillus spp.* DNA, most closely related to *S. moniliformis*. (The data was analyzed with SmartGene software (Lausanne, Switzerland)).

The patient underwent penicillin skin testing, performed by a trained allergy/immunology physician. The skin prick test was administered on the volar forearm with benzylpenicilloyl polylysine (Pre-Pen; ALK) as the major determinant, penicillin G 10,000 U/mL as the minor determinant, histamine 6 mg/mL as the positive control, and sodium chloride 0.9% as the negative control. After a 15-min observation period and with negative skin prick results, intradermal testing was administered using the same materials except with a histamine concentration of 0.02 mg/mL. A positive test result was defined as a wheal ≥3 mm as compared with the negative control.

After testing excluded penicillin allergy, penicillin G 24 mU daily (4 mU every 4 h) was started. She completed 6 weeks of that followed by 2 weeks of oral amoxicillin-clavulanate. During that period, she required brief readmissions for heart failure and dysrhythmias but remained afebrile without signs of infection. Two weeks later she was readmitted for heart failure and fever. One of her pet rats had given birth to a large litter and she reported new rat bite exposures. She was found to have a new 8 mm anterior mitral valve vegetation with valve perforation. She underwent elective dilation and evacuation of the pregnancy to allow for definitive bioprosthetic mitral valve replacement. Blood cultures were persistently negative in the post-operative period, and she was treated empirically for *Streptobacillus*, this time with a six-week course of Penicillin-G and a two-week course of synergistic gentamicin. The repeat mitral valve Gram stain was negative for organisms, and valve fungal, aerobic, and anerobic cultures did not yield any growth.

## Discussion and conclusions

Rat bite fever is a systemic febrile illness caused by infection with the Gram-negative bacillus *Streptobacillus moniliformis* in North America, or the spirochete *Spirillum minus* in Asia, following a bite, scratch, or contact with excrement [1, 2]. A third syndrome is Haverhill fever, caused by ingestion of *S. moniliformis*-contaminated food [1]. Case reports describe exposures among people living in poverty, laboratory technicians, and pet store workers. The affected demographics have broadened, as rats have become more popular pets. *S. moniliformis* colonizes the nasopharynx of 50–100% of healthy wild, lab, and pet rats, and is also excreted in the urine [2]. It is also found in mice, guinea pigs, gerbils, and squirrels. After a bite or scratch, the wound should be immediately cleaned with soap and warm water, and tetanus prophylaxis administered, if warranted. The efficacy of antibiotic prophylaxis for rat bite is unknown. Some authors suggest administration of amoxicillin/clavulanate at a dosage of 500 mg p.o. every 8 h for 3 days.

*S. moniliformis* is a pleomorphic (straight, fusiform, or with lateral bulbar swellings), filamentous, Gram-negative, nonmotile, and non-acid-fast rod [1]. However, on Gram stains it can appear as either Gram-negative or Gram-positive rods.

Rat bite fever typically begins with a bite or other exposure, followed by abrupt onset of systemic illness, including intermittent relapsing fever, arthritis, and rash 3 days to 3 weeks later. A maculopapular, petechial, or purpuric rash develops in approximately 75% of those affected in the first symptomatic week [1, 2]. Over half of those affected develop migratory polyarthralgias, with involvement of both large and small joints of the extremities [1]. If untreated, the mortality rate approaches 10% [2]. Endocarditis is a well-described complication, but only 26 cases of native valve endocarditis have been reported to date [3]. We found no reports involving pregnant patients.

Our patient’s presentation was notably atypical in multiple respects. First, the hyperemesis she experienced was likely wrongly attributed to pregnancy and contributed to delayed diagnosis. Second, she denied a history of rash or arthralgias. Third, eroded intra-oral hardware made fastidious *Streptococcal spp.* or other oral flora equally likely pathogens. Fourth, the diagnosis of the primary infectious agent in this case was further complicated by the positive *Bartonella* IgG titers. *Bartonella* IgG titers between 1:64 and 1:256 represent possible active or recent *Bartonella* infection; our patient’s IgG titers were 1:256. IgM titers > 1:20 strongly suggest current infection; our patients IgM titers were negative. Furthermore, she had no characteristic cutaneous lesions or lymphadenopathy, and there was no *Bartonella* signal detected on the PCR. Taken together, the above essentially rule out *Bartonella* endocarditis. Another confounder was the identification of *Propionibacterium spp.* on the mitral valve specimen. *Propionibacterium spp*. are a very rare cause of infectious endocarditis, and almost always cause prosthetic valve endocarditis. Here, they were most likely a contaminant. Finally, she suffered septic emboli to the right popliteal artery, spleen, and kidneys - a rare complication of rat bite fever endocarditis [4].

Diagnosis of *S. moniliformis* is difficult, requiring a high index of suspicion. It is fastidious, requiring microaerophilic conditions (5–10% CO2 or anaerobic conditions supplemented with 20% normal rabbit serum) [2]. Furthermore, growth is inhibited by 0.05% sodium polyanethol sulfonate (an anticoagulant routinely added to most aerobic blood culture bottles) [1]. When blood cultures remain negative after prolonged incubation, PCR can be used diagnostically. 16sRNA gene sequencing has been used successfully on heart valves, bone, and synovial fluid, but this method is specific only to the *Streptobacillus* genus and not the species [5]. PCR is effective even after antibiotic treatment has been initiated, even if the blood culture is sterile, as the DNA remains detectable in the infected valve.

In this case, blood and valve cultures were persistently negative, despite repeated anaerobic and aerobic sub culturing on various agars and broths including Chocolate, Charcoal Yeast Extract, Columbia, and serum supplemented media. The initial mitral valve specimen was collected surgically 4 days after initiation of empiric antibiotic therapy, likely contributing to the difficulty in culturing the specimen. Broad range bacterial PCR and sequencing of a segment of 16 s rRNA gene matched to *Streptobacillus* sp. (genus level) and most closely related to *Streptobacillus moniliformis* (species level). Speciation of 16 s rRNA gene sometimes can be difficult and erroneous. The patient was counseled about the risks associated with rats especially pertaining to bites.

Recommended treatment of *S. moniliformi*s endocarditis is dual therapy with high-dose penicillin G for 4 weeks in combination with streptomycin or gentamicin for 2 weeks [1, 2]. Ceftriaxone (2gIV daily for 6 weeks) has also been effective [3]. In this case, treatment was limited by the patient’s pregnancy until she underwent dilation and evacuation. Aminoglycosides are pregnancy class D, given several reports of congenital deafness; they are known to cross the placenta.

A literature review was performed by a professional medical librarian using the search strategy presented in the supplemental file. This revealed only two cases, but neither involved endocarditis [6, 7]. One involved a 22 year old woman who developed *Streptobacillus moniliformis* amnionitis [6], and the other involved polymicrobial chorioamnionitis with *Aerococcus christensenii*, *Gemella* spp., *Snethia* spp., *Parvimonas micra*, and *Streptobacillus moniliformis* in a pregnant woman [7].

Our report is limited by the usual features of a single case report, and that more and different samples were not available for duplicate and triplicate laboratory testing. Despite these limitations, it includes the key laboratory and management detail useful for providers who may encounter this in the future, and it appears to be a first reported case based on a thorough literature review described in the supplemental material.

This case highlights the diagnostic and management challenges of an infrequent cause culture negative endocarditis that was further complicated by pregnancy, thromboembolic phenomenon, and a patient’s undaunted love of her pets.

## Data Availability

Not applicable.
